# *In vivo* trafficking of a tumor-targeting IgE antibody: molecular imaging demonstrates rapid hepatobiliary clearance compared to IgG counterpart

**DOI:** 10.1080/2162402X.2021.1966970

**Published:** 2021-09-06

**Authors:** Francis Man, Alexander Koers, Panagiotis Karagiannis, Debra H. Josephs, Heather J. Bax, Amy E. Gilbert, Tihomir S. Dodev, Silvia Mele, Giulia Chiarruttini, Silvia Crescioli, Jitesh Chauhan, Julia E. Blower, Margaret S. Cooper, James Spicer, Sophia N. Karagiannis, Philip J. Blower

**Affiliations:** a School of Biomedical Engineering & Imaging Sciences, King’s College London, London, UK; b School of Cancer & Pharmaceutical Sciences, Institute of Pharmaceutical Science, King’s College London, London, UK; c School of Basic & Medical Biosciences, St John’s Institute of Dermatology, King’s College London, London, UK; d School of Cancer & Pharmaceutical Sciences, Guy’s Hospital, King’s College London, London, UK; e School of Basic and Medical Biosciences, Randall Centre for Cell and Molecular Biophysics, King’s College London, London, UK; f Allergic Mechanisms in Asthma, Asthma UK Centre, King’s College London, London, UK; g Cancer Centre at Guy’s, Guy’s and St Thomas’ NHS Foundation Trust, London, UK; h School of Cancer & Pharmaceutical Sciences, Breast Cancer Now Research Unit, King’s College London, Guy’s Hospital, London, UK

**Keywords:** IgE, IgG, Allergooncology, allergoimmunology, SPECT, nuclear imaging, molecular imaging, cancer immunotherapy

## Abstract

IgE antibodies elicit powerful immune responses, recruiting effector cells to tumors more efficiently and with greater cytotoxicity than IgG antibodies. Consequently, IgE antibodies are a promising alternative to conventional IgG-based therapies in oncology (AllergoOncology). As the pharmacokinetics of IgE antibodies are less well understood, we used molecular imaging in mice to compare the distribution and elimination of IgE and IgG antibodies targeting the human tumor-associated antigen chondroitin sulfate proteoglycan 4 (CSPG4).

Anti-CSPG4 IgE and IgG1 antibodies with human Fc domains were radiolabeled with ^111^In. CSPG4-expressing A375 human melanoma xenografts implanted in NOD-*scid* IL2rg^-/-^ mice were also engrafted with human immune cells by intravenous administration. ^111^In-anti-CSPG4 antibodies were administered intravenously. Their distribution was determined by single-photon emission computed tomography (SPECT) and *ex vivo* gamma-counting over 120 h. SPECT imaging was conducted from 0 to 60 min after antibody administration to precisely measure the early phase of IgE distribution.

^111^In-labeled anti-CSPG4 IgG and IgE showed serum stability *in vitro* of >92% after 5 days. In A375 xenograft-bearing mice, anti-CSPG4 IgE showed much faster blood clearance and higher accumulation in the liver compared to anti-CSPG4 IgG. However, tumor-to-blood and tumor-to-muscle ratios were similar between the antibody isotypes and higher compared with a non-tumor-targeting isotype control IgE. IgE excretion was much faster than IgG. In non-tumor-bearing animals, early SPECT imaging revealed a blood clearance half-life of 10 min for IgE.

Using image-based quantification, we demonstrated that the blood clearance of IgE is much faster than that of IgG while the two isotypes showed comparable tumor-to-blood ratios.

## 1. Introduction

Antibodies have emerged as a major class of drugs in the last two decades, with a variety of a therapeutic and diagnostic applications,^1^ particularly in oncology.^2,3^ Unmodified antibodies can cause cancer cell death by immune mechanisms, such as antibody-dependent cellular cytotoxicity (ADCC) or complement-dependent cytotoxicity (CDC) through specific domains in the antibody Fc region, or independently of the immune system by downregulating cell surface receptors or inducing apoptotic signaling events. Modified antibodies, or bioconjugates, can be used for targeted delivery of drugs, toxins, and diagnostic probes, such as fluorophores and radionuclides.^3^ Both immune and nonimmune activities depend on specific molecular recognition of the target site via the Fab regions of the antibody.^4^ The antibodies used until now for therapeutic or diagnostic applications almost exclusively belong to the IgG class.^1^

**Figure 1.**
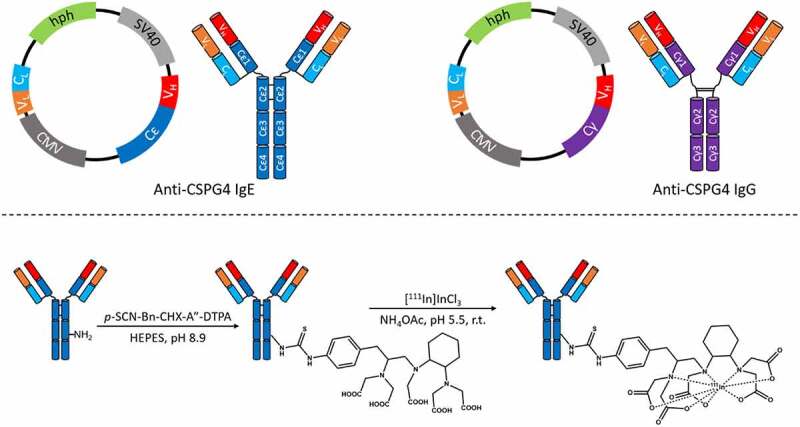
(*Top*) Schematic representation of the cloning strategy employed^6,7^ to produce the human/mouse chimeric anti-CSPG4 IgE and IgG antibodies used in this study. The light chain (V_L_ and C_L_) and the variable region of the heavy chain V_H_ are shared by both antibodies, whereas the Fc parts (Cϵ and Cγ) are of different isotypes. hph: hygromycin B phosphotransferase gene; CMV: human cytomegalovirus enhancer sequence; SV40: Simian Virus 40 enhancer sequence. (*Bottom*) Antibody conjugation and radiolabeling steps. The bi-functional chelator *p*-SCN-CHX-A”-DTPA contains an isothiocyanate functional group that reacts with free amino groups on lysine residues, covalently linking the antibody to the chelator through a thiourea function. The location of the conjugation site in this scheme is for representational purposes only. Diethylenetriaminepentaacetic acid (DTPA) can chelate radiometals such as ^111^In in mild conditions (temperature and pH) that do not lead to antibody degradation

In the search for approaches that might trigger more potent targeted cytotoxic immune responses to tumors, IgE-class antibodies, known for their involvement in powerful immune responses associated with allergic reactions, have shown promise.^5^ This approach exploits the naturally high affinity of the IgE Fc region (Figure 1) to its FcεRI binding site on immune effector cells (monocytes/macrophages, dendritic cells, mast cells, eosinophils).^8–10^ This affinity (K_a_ = 10^10^ M^−1^) is two to five orders of magnitude higher than that of the IgG Fc region for its cognate receptor, FcγRI-III),^11,12^ while the affinity of the IgE Fc for its low-affinity receptor, FcϵRII/CD23 (K_a_ = 10^8^ M^−1^), is similar to that of IgG for FcγRI.^13^ The resulting cross-linking of IgE with its FcϵR receptors has been shown to recruit effector cells to tumors more efficiently than IgG antibodies^14–16^ and consequently evoke greater cytotoxicity and class-specific immune cell-activating and pro-inflammatory signals in the tumor microenvironment,^8,10,15,17–20^ offering the potential to overcome some of the limitations of conventional IgG antibody treatment. In addition to several promising pre-clinical studies, the first clinical trial of an IgE-based immunotherapy (NCT02546921) has shown promising early safety and efficacy data.^21^

Despite the promise of passive IgE anti-tumor therapy in animal cancer models, little is known about the trafficking and fate of IgE *in vivo*. It is known that IgE clears from blood more quickly than IgG, but this difference has not been firmly quantified. The half-life of human IgE circulating in the human serum has been estimated to be in the range up to 3 days,^22,23^ whereas for IgG a serum half-life of up to 23 days^23–26^ has been estimated. In mice, these values range from 1.5 to 14 h for IgE, compared to 6–10 days for IgG.^27–30^ Conversely, estimates of residence time of IgE in tissues (mainly skin) suggest a much longer residence time for IgE (7–14 days in rats,^31,32^ 20 days in humans^33^) compared with a shorter tissue retention for IgG (2–3 days). The fate and transport mechanisms of IgE after clearance from circulation have not been fully elucidated.

In this study, we use radionuclide molecular imaging, for the first time, to compare the trafficking and biodistribution of two engineered homologous IgG and IgE antibodies^6,7^ targeting a tumor-associated antigen, CSPG4 (chondroitin sulfate proteoglycan 4): anti-CSPG4 IgG and anti-CSPG4 IgE. These antibodies, respectively, have a human IgG and IgE Fc region but share the same mouse variable region targeted to the CSPG4 antigen (Figure 1).^34^ CSPG4 is expressed in a number of normal tissues and pluripotent progenitor cell populations,^35^ taking part in angiogenesis and stem cell motility.^36,37^ CSPG4 is of great interest clinically because it is highly expressed in >80% of primary and metastatic melanoma lesions^35,38,39^ and its expression has been correlated to resistance of melanoma to conventional chemotherapy.^40^ CSPG4 expression is also associated with various cancers other than melanoma including oligodendrocytomas, gliomas, triple-negative breast carcinomas, squamous cell carcinoma, and lymphoma.^39,41,42^ It has been successfully used as a marker to locate melanoma lesions with radiolabeled IgG monoclonal antibodies (mAb),^43,44^ and as a target for IgG1- and CAR-T-based immunotherapy.^38,45–47^ We have recently demonstrated the *in vivo* safety of a rat IgE targeting CSPG4 in immunocompetent animals.^48^ By radiolabeling each of the antibodies with ^111^In (Figure 1) and using SPECT imaging in mice, we aimed to provide a more precise and complete picture of the *in vivo* fate and pharmacokinetic behavior of an anti-tumor IgE therapeutic candidate^34^ compared to its IgG counterpart.

## 2. Materials and methods

### Ethics

Animal experiments were approved by the UK Home Office under The Animals (Scientific Procedures) Act (1986), with local approval from King’s College London Research Ethics Committee (KCL-REC). Mice were maintained under specific pathogen-free conditions. Experiments using human cells received approval from KCL-REC. All donors provided written, informed consent.

### Anti-CSPG4 antibody conjugation and radiolabeling

Anti-CSPG4 IgE and IgG antibodies were engineered and produced as previously described.^6,7^ Further details of anti-CSPG4 IgE and IgG production and conjugation are provided in the Supplementary Material. Briefly, anti-CSPG4 antibodies (8–10 mg/mL in 0.1 M HEPES, pH 8.9) were incubated overnight at 4°C with a 20 × molar excess of *p*-SCN-CHX-A”-DTPA (Macrocyclics). The antibodies were buffer-exchanged into 0.2 M ammonium acetate buffer (pH 6) and concentrated to 2–3 mg/mL.

Indium-111 (30–180 MBq) in 60–300 µL of 0.1 M hydrochloric acid (Curium, UK) was added to the DTPA-conjugated antibody (200–700 μg at 2–3 mg/mL in 0.2 M NH_4_OAc, pH 6) and incubated for 30 min at room temperature. Labeling efficiency was measured by radio-thin layer chromatography (radio-TLC) and high-performance liquid chromatography (radio-HPLC). Radio-TLC was performed on ITLC-SA paper strips (Varian) with a mobile phase of 0.1 M sodium citrate (pH 5) with 5 mM EDTA. The strips were analyzed using a Mini-Scan™ radioTLC linear scanner (LabLogic Systems) equipped with a gamma probe (LabLogic B-FC-3200). Radio-HPLC was performed on an Agilent 1200 system using a size-exclusion chromatography column (BioSep SEC-s2000, 300 × 7.8 mm, 5 μm particle size, 145 Å pore size; Agilent) and phosphate-buffered saline pH 7.4 containing 0.2 mM EDTA as mobile phase (1 mL/min). Signals were detected with a G1314B UV detector (Agilent) and a gamma probe (LabLogic B-FC-3200). As radiolabeling efficiencies of >94% were found, no post-labeling purification was required.

Stability of the antibody conjugates in serum was assessed by incubating 245 μg of antibody radiolabeled with 30–31 MBq ^111^In in 1 mL of human AB type serum (Sigma) at 37°C. Samples were analyzed by radio-HPLC at 0 and 120 h. Stability was calculated as the AUC of the antibody peak as a percentage of the total activity.

### In vitro *binding of anti-CSPG4 antibody conjugates to CSPG4 and Fc receptors*

*In vitro* binding of anti-CSPG4 antibodies to U937 human monocytic cells (expressing Fcγ and Fcϵ receptors) and A375 human melanoma cells (expressing CSPG4) was assessed by flow cytometry, and immunofluorescence microscopy for A375 cells. Briefly, cells were incubated with non-radiolabeled DTPA-analog conjugated anti-CSPG4 IgE and IgG. The primary antibodies were detected using goat anti-human IgE-FITC or IgG-FITC (Jackson ImmunoResearch). Flow cytometry analysis was performed in FACSCalibur™ or FACSMelody™ (BD Biosciences) instruments. Immunofluorescence images were acquired on an Eclipe Ti inverted microscope (Nikon). Further details are provided in the Supplementary Material.

### A375 melanoma model with human leukocyte engraftment

Male and female NOD-*scid* IL2rg^-/-^ mice (NSG, NOD.Cg-Prkdc*^scid^* Il2rg*^tm1Wjl^*/SzJ; The Jackson Laboratory) were used at between 6 and 10 weeks of age, with equal numbers of males and females in each group. Human PBLs were isolated and implanted as previously described.^49^ Briefly, approximately 5 × 10^5^ A375 melanoma cells were injected subcutaneously into the lower left flank. Five days later, heparinized human peripheral blood (25 mL) was haemolysed using ammonium-chloride-potassium (ACK) lysis buffer. The remaining PBLs were isolated by centrifugation, washed twice in PBS and injected intravenously to the mice (1 × 10^7^ PBL/mouse). Tumor size was measured with callipers and volume calculated using formula: V = d^2^ × (D/2), where d = small diameter and D = large diameter. Spleen engraftment of human CD45^+^ cells was confirmed by flow cytometry.^34^ A therapeutic study of the anti-CSPG4 IgE in this xenograft model is described in Chauhan *et al*.^34^

### In vivo *SPECT imaging of anti-CSPG4 IgE and IgG, biodistribution studies*

SPECT/CT imaging was performed on a NanoSPECT/CT scanner (Mediso; 1.0 mm collimators) in helical scanning mode using energy windows centered around 171.3 keV and 245.4 keV (±20%). For t = 0–60 min, images were acquired in six 10-min windows using 13 s/projection. At t = 4, 24 and 120 h, images were acquired over 50–55 min using 70 s/projection. CT images were acquired using 45 kVp tube voltage and 1200 ms exposure time in 180° projections. Mice were anaesthetized with isoflurane and ^111^In-labeled anti-CSPG4 IgE or IgG (70 μg/mouse i.e. 2.5 mg/kg, 20 MBq ^111^In, n = 4) were administered intravenously at t = 0 h, immediately after starting the scan. SPECT/CT data sets were reconstructed using a Monte-Carlo-based full-3D iterative algorithm (Tera-Tomo, Mediso). Images were co-registered and analyzed using VivoQuant v2.50 (Invicro). Regions of interest (ROIs) were delineated for ^111^In activity quantification in specific organs. Uptake in each ROI was expressed as percentage of injected dose per volume (%ID/mL).

Mice for *ex vivo* biodistribution studies at t = 4 h (n = 4) and 24 h (n = 4) were administered ^111^In-labeled anti-CSPG4 IgE or IgG (70 μg/mouse, 3 MBq ^111^In) intravenously. At t = 120 h, *ex vivo* biodistribution studies were performed with the mice used for imaging. Mice were culled by cervical dislocation, organs were dissected, weighed, and gamma-counted along with standards prepared from a sample of injected antibody. Antibody uptake was calculated as a percentage of injected dose per gram (%ID/g) of tissue. Blood clearance rates were calculated using a one-phase exponential decay equation.

Excretion of indium-111 was calculated by deducting the sum of the radioactivity in organs, tissues, and carcass from the total injected activity, after decay correction. Excretion rates were calculated using a two-phase exponential decay equation, constrained to a final plateau value of zero and a starting value of 100%.

### Statistics

Statistical analyses were performed in GraphPad Prism version 9. Differences between IgE, IgG, and control groups were analyzed by Student’s t-test or ANOVA testing, as appropriate.

## 3. Results

### Antibody production and radiolabeling

The anti-CSPG4 IgE and IgG antibodies (Figure 1) were produced as described in the Supplementary Material. After antibody conjugation to the bifunctional chelator *p*-SCN-CHX-A”-DTPA, SDS-PAGE analysis revealed bands corresponding to full-size IgG and IgE^6^ (Fig. S1) and, under reducing conditions, corresponding to the IgG and IgE heavy and light chains.

The conjugation of the chelator was successful, as evidenced by ^111^In-radiolabeling efficiencies of 96.9% for the IgG and 92.7% for the IgE (Fig. S2A). Further purification was judged unnecessary. Non-antibody-bound radioactivity (<8%) can be seen eluting as EDTA complexes with a retention time of 12.8 min. Similar results were obtained by radio-TLC, with the radiolabeled antibody conjugates remaining at the origin whereas free ^111^In was chelated by citrate ions in the mobile phase (R_f_ = 0.8; Fig. S3). The radio-HPLC traces after 120 h incubation in serum showed some signs of degradation of the radiolabeled antibody, with increased area under the curve (AUC) of peaks corresponding to a higher molecular weight impurity and to unchelated ^111^In. The degradation was more pronounced for the IgE (decrease in radiochemical purity from 92.7% to 82.7%) than the IgG (decrease from 96.9% to 92.8%, Fig. S2B).

### In vitro *targeting of CSPG4*

**Figure 2.**
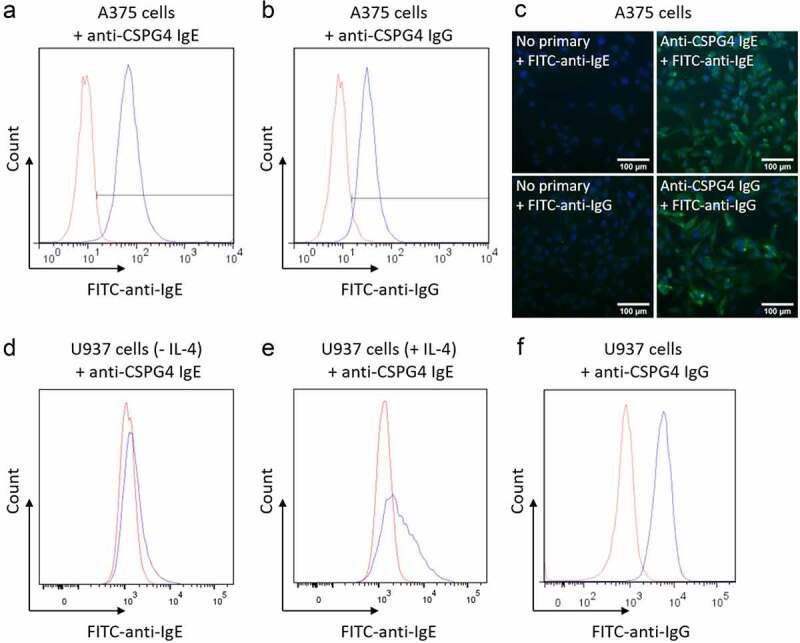
***In vitro* evaluation of antibody binding to target**. (a,b) Representative histograms and (c) immunofluorescence microscopy images (20 × magnification) showing the binding of anti-CSPG4 IgE and anti-CSPG4 IgG conjugates to CSPG4-overexpressing A375 cells. For immunofluorescence microscopy, A375 cells (nuclei in blue) were incubated with or without anti-CSPG4 IgE and IgG conjugates, followed by FITC-labeled anti-human-IgE or anti-human-IgG secondary antibodies, respectively. Scale bars represent 100 μm. (d,e) Representative histograms showing the binding of anti-CSPG4 IgE to Fcε receptors on U937 cells primed with IL-4 (10 ng/mL) to induce FcεRII/CD23 expression. (f) Representative histogram showing the binding of anti-CSPG4 IgG to FcγRI on U937 cells. Blue lines represent the anti-CSPG4 Ig conjugates detected with a secondary AF488 antibody. Red lines show the secondary antibody alone

The binding of the conjugated antibodies via the Fab region to the target antigen CSPG4 on tumor cells was evaluated by flow cytometry and immunofluorescence microscopy. The binding of the conjugated antibodies via the Fc to their cognate Fc receptors on immune cells was evaluated by flow cytometry. Both the IgE and IgG showed comparable binding characteristics to CSPG4^high^ A375 cells (Figure 2a-c). Binding of the anti-CSPG4 IgE to U937 monocytic cells was observed after priming with IL-4 to induce FcϵRII expression (Figure 2c,d), suggesting this IgE conjugate binds to its cognate Fcϵ receptors in line with previous reports.^6^ Similarly, binding of the anti-CSPG4 IgG to U937 cells was observed, suggesting recognition of the conjugated antibody by the FcγRs present on these monocytic cells (Figure 2e).^47^ These findings suggest that the *p*-SCN-CHX-A”-DTPA conjugated antibodies could bind to target antigen-expressing and Fc receptor-expressing cells.

### In vivo: *pharmacokinetic behavior and half-life determination*

To study the biodistribution of anti-CSPG-IgE, a previously described mouse model of melanoma with a humanized immune system was employed.^49^ A detailed study of the anti-tumor efficacy of the anti-CSPG4 IgE was recently performed by Chauhan *et al*., showing that this antibody significantly delayed the growth of A375 tumors, resulted in increased macrophage recruitment to tumor tissue and prolonged survival of mice bearing patient-derived melanoma xenografts.^34^

**Figure 3.**
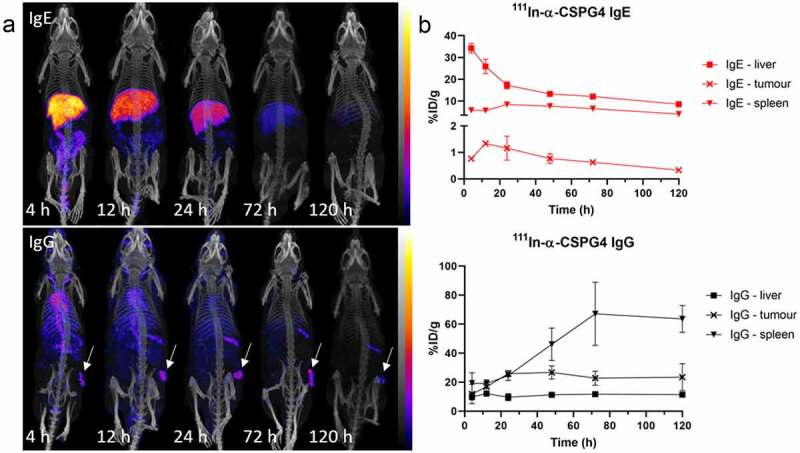
**SPECT/CT imaging and biodistribution of anti-CSPG4 IgE/IgG in A375 melanoma model**. (a) Representative SPECT/CT images (maximum intensity projections) of ^111^In-labeled anti-CSPG4 IgE (top) and anti-CSPG4 IgG (bottom) at 4 h, 12 h, 24 h, 72 h and 120 h after i.v. administration (70 µg antibody, 20 MBq ^111^In) in a subcutaneous A375 melanoma xenograft tumor model with spleen-engrafted human PBLs. The images show that the IgE is mostly found in the liver after only 4 h, and some intestinal excretion taking place. In contrast, the IgG is visible in the large blood vessels for over 12 h, as well as in the tumor and spleen throughout the experiment. Arrows indicate tumor location. (b) Uptake of ^111^In-labeled anti-CSPG4 IgE (top) and anti-CSPG4 IgG (bottom) in the liver, spleen and tumors at 4 h, 12 h, 24 h, 72 h and 120 h after administration (70 µg antibody, 3–4 MBq ^111^In). Symbols represent the mean ± SD of n = 3 animals per group (n = 2 for anti-CSPG4 IgG and MOv18 IgE at 12 h and 120 h, n = 4 for anti-CSPG4 IgG at 72 h), error bars smaller than the symbols are not depicted

**Figure 4.**
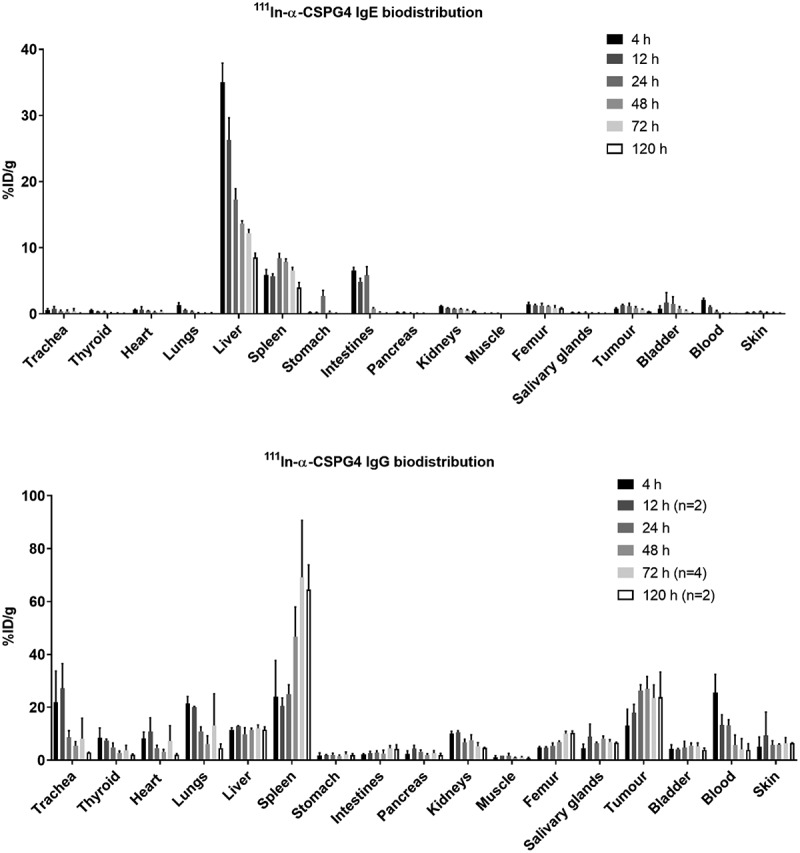
**Biodistribution of ^111^In-labeled anti-CSPG4 IgE (top) and anti-CSPG4 IgG (bottom)** at 4 h, 12 h, 24 h, 48 h, 72 h and 120 h after i.v. administration in a subcutaneous A375 melanoma xenograft tumor model with splenic engrafted human PBMCs, showing the uptake of ^111^In in each organ. Notably, the IgE mostly accumulates in the liver, spleen and intestines, whereas the IgG distributes more broadly and remains in the blood in much higher amounts. Values determined by gamma-counting. Uptake in each organ is expressed as %injected dose per gram (%ID/g), bars represent the mean ± SD of n = 3 (unless otherwise indicated) animals per time point

Radiolabeling of anti-CSPG4 IgG/IgE allowed a longitudinal study of the distribution and pharmacokinetics of these antibodies in the mouse tumor model by SPECT imaging. Differences in distribution between the IgE and IgG are clearly visible in the SPECT/CT images (Figure 3a). The IgG showed a biodistribution and blood clearance typical of radiolabeled IgG antibodies used in many nuclear medicine applications in recent decades: slow blood clearance with an estimated half-life of several days (as evidenced by radioactivity visible in the heart and major vessels) and distinct accumulation in tumor and spleen at later time points but no marked uptake in other tissues. The IgE, in contrast, was rapidly cleared from the circulation and accumulated in the liver and to some extent in the kidneys. Later, IgE radioactivity levels decreased somewhat in liver and increased in the intestines. The tumor was not readily visible in the scans at any time point after ^111^In-IgE administration. The tumor uptake of ^111^In-anti-CSPG4 IgG was clearly visible and reached a plateau of 16–30%ID/g between 24 h and 72 h, the spleen uptake peaked at 67 ± 21%ID/g at 72 h, and liver uptake was relatively constant at 9–11%ID/g between 4 h and 120 h (Figure 3b). In contrast, uptake of ^111^In-labeled IgE in the tumor showed a peak 12 h after administration at around 1.5%ID/g, spleen uptake remained between 4% and 8%ID/g throughout, and liver uptake was initially very high at 34%ID/g, progressively decreasing to 8.5%ID/g at 120 h. The imaging results were confirmed with quantitative analysis by *ex vivo* gamma-counting of individual organs at intervals between 4 h and 120 h post injection (Figure 4).

**Figure 5.**
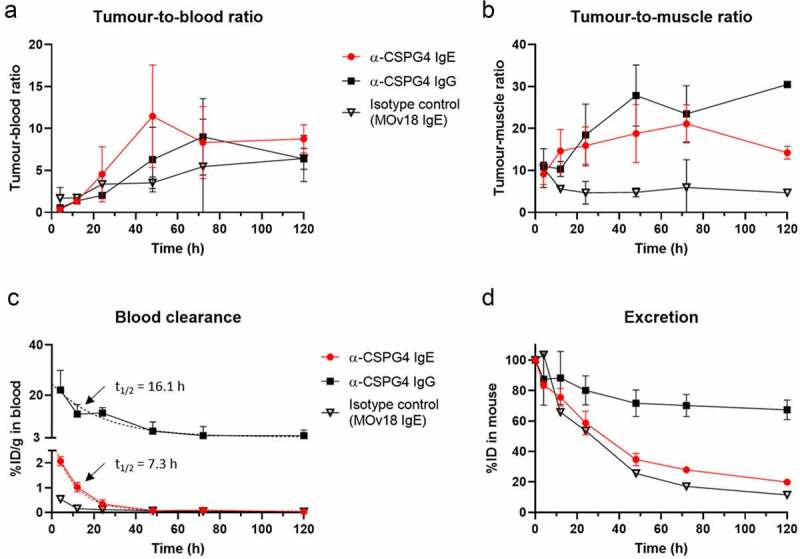
**Tumor accumulation, blood clearance and excretion of anti-CSPG4 IgE/IgG in A375 melanoma model**. (a) Tumor-to-blood and (b) tumor-to-muscle (right) ratios of ^111^In after administration of ^111^In-labeled anti-CSPG4 IgE, anti-CSPG4 IgG or isotype control antibody (MOv18 IgE), showing similar trends between the IgE and IgG over time, despite large differences in tumor uptake. Ratios were calculated based on %ID/g values determined by gamma-counting dissected organs. (c) Blood clearance, showing much faster clearance of both IgE antibodies compared to the IgG antibody. NB: in this case, sampling was started at t = 4 h, meaning that the early clearance phase was missed and resulting in a misleading initial estimate of half-life for the IgE. Each point represents the percentage of injected ^111^In present in the blood, based on gamma-counting data and total blood volume. (d) Antibody excretion over time. Each point represents the percentage of ^111^In remaining in the mouse after administration of ^111^In-labeled anti-CSPG4 IgE, anti-CSPG4 IgG or and nonspecific IgE (MOv18) at t = 4 h, 12 h, 24 h, 72 h and 120 h after administration (70 µg antibody, 3–20 MBq ^111^In), determined by gamma-counting dissected organs. Symbols represent the mean ± SD of n = 3 animals per group (n = 2 for anti-CSPG4 IgG and MOv18 IgE at 12 h and 120 h, n = 4 for anti-CSPG4 IgG at 72 h); error bars smaller than the symbols are not depicted. Dashed lines represent curve fits

Despite marked difference in absolute tumor uptake of the two antibodies, their tumor-to-blood (T/B) and tumor-to-muscle (T/M) ratios were similar (T/B: IgE: 11.5 at 48 h *c.f*. IgG: 9.0 at 72 h; T/M: IgE: 21.1 at 72 h *c.f*. IgG: 27.8 at 48 h) (Figure 5a,b). In contrast, a control MOv18 IgE antibody, targeted toward a different antigen, folate receptor-alpha, which is not expressed by A375 melanoma cells, and used here as a nonspecific isotype control, showed much lower T/B and T/M ratios than those of the anti-CSPG4 antibodies at 24–120 h (Figure 5a,b). These results suggest that both anti-CSPG4 IgG and anti-CSPG4 IgE displayed similar and specific affinity for the CSPG4-expressing tumor and that differences in tumor accumulation may be due to other factors affecting their pharmacokinetics.

The initial half-life of ^111^In-anti-CSPG4 IgG in the blood was calculated as 16.1 h (single-phase exponential decay, least-squares regression: R^2^ = 0.7355) with a plateau at 3.3%ID/g, whereas ^111^In-anti-CSPG4 IgE was cleared from the circulation much faster with a calculated blood half-life of 7.3 h (R^2^ = 0.9778, plateau value: 0.05%ID/g) based on samples taken between 4 h and 120 h (Figure 5c). The apparently good fit to a mono-exponential decay curve with near-zero plateau value for the IgE is misleading and suggests that the initial rapid distribution phase was missed and occurred earlier than 4 h.

The excretion of ^111^In was estimated based on the total activity in the mice over time. For the anti-CSPG4 IgE, 84 ± 2% of the administered activity remained in the mice after 4 h, dropping to 59 ± 8% after 24 h and 20 ± 1% after 5 days. The isotype control IgE followed a similar curve, with 54 ± 9% remaining after 24 h and 11 ± 1% after 5 days. For the anti-CSPG4 IgG, 87 ± 17% of the injected activity was still present after 4 h and excretion was much lower throughout the experiment, with 80 ± 9% remaining after 24 h and 67 ± 6% after 5 days (Figure 5d). These results, combined with the imaging, suggest that the anti-CSPG4 IgE is rapidly eliminated through the liver.

**Figure 6.**
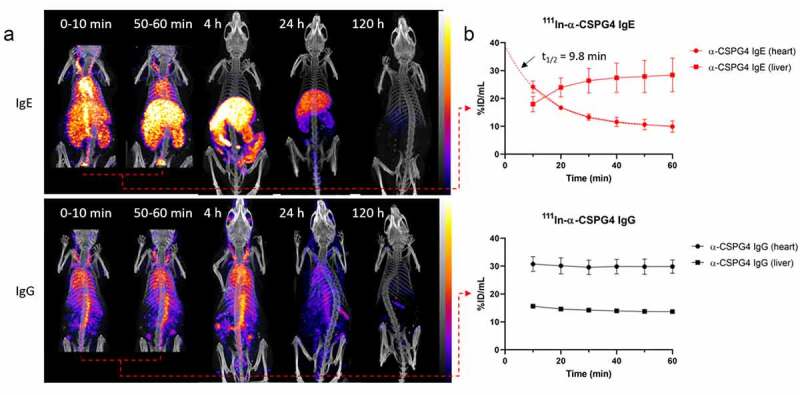
**SPECT/CT imaging and quantification of anti-CSPG4 IgE/IgG in non-tumor-bearing mice**. These data add to those shown in Figure 5 by including the early-phase distribution and clearance. (a) Representative SPECT/CT images (maximum intensity projections) of ^111^In-labeled anti-CSPG4 IgE (top) and anti-CSPG4 IgG (bottom) at 0–10 min, 50–60 min, 4 h, 24 h and 120 h after i.v. administration (70 µg antibody, 20 MBq ^111^In) in NSG mice with spleen-engrafted human PBLs, showing the rapid clearance of the IgE from the circulation and the much longer circulation of the IgG. A narrower field of view was used in the first hour to reduce overall scan times without reducing sensitivity (frame acquisition time). (b) Image-based quantification of ^111^In-labeled anti-CSPG4 IgE (top) and anti-CSPG4 IgG (bottom) in blood (heart) and liver over the first 60 min administration. Symbols represent the mean ± SD of n = 4 animals per group; error bars smaller than the symbols are not depicted. Dashed lines represent curve fits

Noting the rapid blood clearance of anti-CSPG4 IgE based on the biodistribution and imaging data in tumor-bearing mice, we suspected that the first imaging and *ex vivo* biodistribution measurements taken at 4 h were too late to provide an accurate estimate of the true half-life of the early clearance phase and instead overestimated the half-life. The imaging experiment was therefore repeated, with earlier scanning, in mice with a partially reconstituted human immune system but without tumors (since the aim in this case was to determine blood clearance half-life and not tumor targeting). To capture the early distribution of the antibodies, SPECT images were acquired in blocks of 10 min over the first hour, starting immediately from antibody injection. These earlier scans revealed that the anti-CSPG4 IgE cleared more rapidly from the circulation than calculated from the previous experiments; high uptake was seen in the liver and kidneys in the first 10 min after administration and very little circulating radioactivity was observed 50–60 min after administration (Figure 6a). By fitting the blood radioactivity values in the heart region to a one-phase exponential decay function, anti-CSPG4 IgE was determined to have an initial blood half-life of *t*_1/2 _= 9.8 min (R^2^ = 0.92) (Figure 6b). In contrast, in a similar experiment with the IgG, radioactivity was mostly visible in large blood vessels, lungs, and spleen at those time points. In female mice, anti-CSPG4 IgG uptake in the ovaries was also observed. The time-activity curve for anti-CSPG4 IgG in blood appeared to be near-constant over the first hour and the clearance of this IgG is perhaps better represented in Figure 4b. Between 4 h and 120 h after administration, uptake profiles of the antibodies in various organs were qualitatively similar to those in the tumor-bearing animals, with high accumulation in the liver and low accumulation in the blood and spleen for anti-CSPG4 IgE, and higher persistence in the circulation and increasing accumulation in the spleen for anti-CSPG-IgG (Fig. S4).

## 4. Discussion

Although the use of IgE-class antibodies in anti-cancer therapy has been the focus of many pre-clinical studies (recently reviewed by Sutton *et al.*,^5^ Chauhan *et al*.^50^), the fate and distribution of exogenous IgE-based drugs after administration remains unclear. By comparing an IgE and an IgG antibody sharing the same CSPG4-targeting variable domains, we aimed to determine the impact of these different immunoglobulin backbones on *in vivo* antibody distribution and uptake in CSPG4-expressing tumors. Having conjugated the antibodies to a DTPA-analogue chelator for radiolabeling with the gamma-emitting radionuclide ^111^In (*t*_1/2 _= 67.3 h), we used SPECT/CT imaging to perform longitudinal, noninvasive, and quantitative measurements of antibody distribution. The long half-life of ^111^In is appropriate to track large biomolecules with long biological half-lives. Whilst limited degradation was noted after 5 days incubation in serum, a large majority of the ^111^In remained bound to the antibodies, an encouraging sign that the ^111^In signal observed *in vivo* would primarily arise from the antibody conjugates or their metabolites rather than from “free”, de- or trans-chelated ^111^In.

A key element when labeling a compound for imaging studies is that the reporter moiety should, ideally, not affect the biological properties of the parent compound. We found that the anti-CSPG4 IgE and IgG antibodies in their conjugated forms could bind to CSPG4-overexpressing A375 melanoma cells and to Fc receptor-expressing immune cells. Although the priming of U937 cells with IL-4 was required to induce significant expression of the low-affinity FcϵRII receptor for IgE binding in this model *in vitro*, tumor cell killing by IgE-mediated ADCC does not require administration of IL-4.^15,34^ Whether IgE immunotherapy may be primed in Th2 environments requires further investigation. The radiolabeled anti-CSPG4 IgG/IgE conjugates were thus shown to be suitable candidates for *in vivo* imaging studies. Importantly, we observed in the A375 xenograft tumor model that the T/B and T/M ratios were similar for both antibody isotypes and much higher for specific antibodies compared with a non-tumor targeted control IgE, demonstrating that target-binding capability was preserved *in vivo.*

A limitation of studying human IgE-class antibodies in rodent models is the very low affinity of these antibodies for rodent Fcϵ receptors.^11^ Consequently, they do not elicit the same immune response as in humans. To overcome this, we used an established mouse model with a partially reconstituted human immune system,^49,51^ in which the administration of human peripheral blood immune cells leads to durable engraftment of human leukocytes, including of monocytic cells, in the spleen as well as in A375 melanoma xenografts in NSG mice. In this model, the anti-CSPG4 IgE antibody significantly restricted the growth of A375 tumors and patient-derived xenograft tumors compared to the nonspecific IgE control antibody and significantly greater macrophage infiltration into tumor tissues was observed in animals treated with anti-CSPG4 IgE.^34^ An alternative would be to use an immunocompetent model and surrogate antibodies engineered to be of the same host species. We have previously reported the development of anti-CSPG4 IgE and anti-Folate Receptor alpha IgE antibodies engineered with rat Fc regions and we have evaluated the safety of rat anti-CSPG4 IgE in immunocompetent rat models.^15,48,52^ Pharmacokinetic studies of rat IgE in immunocompetent models will be the subject of future work. While a detailed investigation of the immunological mechanisms underlying the effects of anti-CSPG4 IgE was not the objective of the present study, it was previously demonstrated that IgE signaling could recruit and activate macrophages through a TNFα/MCP-1 pathway to adopt a pro-inflammatory, anti-tumoral phenotype, alongside triggering immune signaling pathways signifying parasite clearance rather than allergic response.^15,16,52,53^ Importantly, we have shown that a human/mouse chimeric IgE did not cause basophil degranulation in blood samples from cancer patients,^34,54^ and that the rat anti-CSPG4 IgE was shown to be safe in an immunocompetent rat model.^48^

As the prospect nears of using engineered IgE antibodies in cancer therapy, a better understanding of the pharmacokinetic behavior of this antibody class becomes increasingly necessary. A striking aspect of IgE antibodies is their rapid clearance from circulation compared to IgG. Published estimates of their half-life in circulation range from 1–2 h to 5–16 h (short and long half-life components of a bi-exponential clearance) in mice,^27–30^ 12 h in rats^32^ and 2–3 days in humans.^22,23,33,55,56^ The rate of clearance has also been expressed as fractional catabolic rate (FCR) per day, with FCR values for human IgE ranging from 20% to >90% per day.^22,23,55^ In comparison, serum half-lives of 23 days and FCR of 4–8% have been found for human IgG.^23–26^ Considering our present results, we suggest that methodological issues (lack of very early sampling) in some of these studies may have led to significant overestimations of IgE half-life and the real values are likely even shorter than has been reported. For example, in studies performed in the late 1970s, patients were infused with IgE-rich serum over 4 h and the initial sampling performed 1 h after infusion was taken as the peak IgE serum concentration.^33,56^ In some studies in mice, the explicit assumption was made that the entire injected dose of ^125^I-labeled IgE was still in the serum 10 min after injection.^22,23^ In rats, Tada *et al*. first sampled 15 min after injection of IgE and then at 12 h intervals.^32^ Vieira and Rajewski found an IgE half-life in serum of 7–16 h, however, their initial sampling appears to have been performed 6 h after administration.^29^ In the study by Hirano *et al*., the initial sampling was performed either 15 min or 1 h after administration, resulting in both cases in estimated half-lives of around 12 h. Finally, Haba *et al*. usefully distinguished between an earlier phase and a later phase for their *t*_1/2_ calculations, with an apparent half-life of around 1.5–2 h in the first six hours and 5–8 h over the following days.^28^ In our initial experiment in tumor-bearing mice, primarily aimed at comparing antibody uptake in the tumors, we initially sampled 4 h after administration and found an apparent blood half-life of 7.3 h for the anti-CSPG4 IgE, in line with the aforementioned studies. The SPECT images of tumor-bearing mice, however, qualitatively suggested a much more rapid clearance of the administered IgE from the circulation. To focus on the early pharmacokinetics of IgE, we repeated the experiments in non-tumor-bearing mice with an imaging protocol refined by adding several early time points, allowing us to determine that anti-CSPG4 IgE had in fact an initial blood half-life of less than 10 min, which is much shorter than previously reported.

Imaging the animals from the point of antibody administration revealed that even in the first 10 min, a large fraction of the administered IgE had already cleared from the circulation and was found in the liver and kidneys, contrary to previous assumptions that early blood sampling represented the entire administered dose. Quantitative whole-body imaging conveniently allows measurement of the accumulation and clearance of the administered immunoglobulin on an organ-by-organ basis, which was not possible in previously reported studies of IgE pharmacokinetics. On the other hand, the SPECT signal represents the radionuclide and cannot distinguish the parent antibody from its metabolites, whereas techniques such as the radioimmunoassays used in earlier studies can quantify a relatively intact antibody. Waldmann, Iio and colleagues suggested the existence of two catabolic pathways, one common to all immunoglobulins and one specific to IgE.^22^ Using ^125^I- and ^131^I-labeled IgE, they later demonstrated that the high catabolic rate of IgE was in significant part due to an extravascular pathway specific to this antibody class, and suggested that it occurred through membrane-bound proteases on the surface of FcϵRI-bearing cells, such as basophils and mast cells.^23^ While we observed some early presence of ^111^In in the kidneys, which could be due to the presence of unchelated ^111^In, the high and rapid uptake of the IgE in the liver suggests that instead, the liver is the main location for IgE catabolism, followed by fecal excretion of radioactive metabolites as visible on SPECT images. By using total body radioactivity measurements, we also determined that ^111^In-anti-CSPG4 IgE (and its radioactive metabolites) was eliminated from the body much faster than its IgG counterpart. This may be due to differences in glycosylation patterns between IgE and IgG. Indeed, human IgE carries 7 *N*-glycosylation sites per heavy chain, characterized by the presence of terminal oligomannose and galactose residues, compared to a single *N*-glycosylation site on human IgG.^57^ These residues enable the recognition of IgE by sugar-binding receptors such as the mannose receptor and asialoglycoprotein receptors, which are expressed on liver cells and mediate the rapid clearance of mannosylated and galactosylated proteins.^58^ Furthermore, recent studies have shown that in the human PBL engraftment model we used, human CD11b^+^ (monocytes, macrophages, NK cells) and CD11c^+^ cells (monocytes, dendritic cells) are found in relatively high numbers in the liver and spleen,^59^ and that human FcϵRI expressed on dendritic cells and monocytes are responsible for the internalization, degradation, and rapid clearance of human IgE in humanized mice.^60^ Our results are consistent with these previous studies. In contrast to IgE, the anti-CSPG4 IgG showed a distribution and clearance rate typical of labeled monoclonal IgG antibodies, with long persistence in the circulation and accumulation in the spleen, which can be explained by the binding of the IgG to Fcγ receptors expressed on lymphocytes engrafted in the spleen. The scarcity of signal originating from the kidneys is in accordance with the high stability shown by the IgG in serum. Despite inconsistencies in literature reports of sampling times, amounts, and purity of IgE administered (70 μg to 1.5 mg, affinity-purified or IgE-rich serum) and differences in antigen specificity, models, and assay techniques, there is a consensus that the short serum half-life is an intrinsic property of IgE. Lack of a domain for recognition of the neonatal Fc receptor (FcRn) on the IgE structure^61^ and differences in the glycosylation patterns of IgE compared to IgG^5^ may provide a potential explanation for this phenomenon.

We report that despite the fast clearance of IgE from the blood, the tumor-to-blood ratios of anti-CSPG4 IgE were much higher than those of a non-tumor targeted IgE, suggesting that anti-CSPG4 IgE could reach its target antigen in tumors. Using the same model of A375 tumors, we recently demonstrated that anti-CSPG4 IgE restricted tumor growth *in vivo* compared to a nonspecific isotype control IgE.^34^ IgE immunotherapy was associated with enhanced tumor infiltration and activation of monocytes and macrophages with pro-inflammatory phenotype characteristics. Although in our study the radiolabeled antibodies were administered at doses (2.5 mg/kg) well below those required to confer therapeutic effects (10 mg/kg) in these models, our findings point to a scenario where a relatively small proportion of administered IgE reaching the tumor might be sufficient to activate immune effector cells and potentially confer anti-tumor effects. This is consistent with known properties of IgE-immune complexes to trigger strong effector cell activation when bound to cell surface FcεRs at levels well below saturation. Early data from the first clinical trial of MOv18 IgE corroborate our results, as both rapid plasma clearance of the IgE and reduction in the tumor biomarker CA-125 were observed.^21^ As a practical consequence, blood sampling in patients may not be an ideal guide for dosing IgE-based therapies, and typical immunotherapy dosing schedules consisting of a loading dose followed by maintenance doses may not be appropriate. Instead, determining the amount of drug reaching the tumors of human subjects, for which nuclear imaging is a powerful tool,^62^ may be a preferable approach. Future imaging studies should be performed in the context of clinically relevant IgE doses, as the presence of a liver sink for the IgE means that IgE biodistribution and tumor uptake may be different if the Fc receptors are saturated. Considering the potent immune responses triggered by IgE antibodies, imaging biomarkers of immune cell recruitment and activation status in the tumor micro-environment may also be more appropriate indicators of the pharmacodynamic attributes of this therapeutic class.

In summary, by radiolabeling both an IgE and an IgG targeting the tumor-associated antigen CSPG4, we have demonstrated that the blood half-life of IgE antibodies is, at around 10 min, much shorter than previously published and that the radioactivity delivered by IgE antibodies is mostly eliminated through the liver followed by excretion via the intestinal tract. In spite of rapid clearance, IgE does reach and is retained in tumors and the tumor-to-blood ratios for IgE and IgG antibodies are comparable. These marked pharmacokinetic differences between IgE and IgG point to differential mechanisms by which each antibody isotype may operate in the context of cancer therapy.

## Supplementary Material

Supplemental Material
