# Supplementary material for: In vivo trafficking of a tumor-targeting IgE antibody: molecular imaging demonstrates rapid hepatobiliary clearance compared to IgG counterpart
Source: Oncoimmunology. 2021 Sep 6;10(1):1966970. doi: 10.1080/2162402X.2021.1966970 (PMC8425638; doi:10.1080/2162402X.2021.1966970)
Supplement: Supplemental Material [file KONI_A_1966970_SM4747.zip › IgE_paper_FMA_supplement_revision1_CLEAN.docx]

*In vivo* trafficking of a tumour-targeting IgE antibody: molecular imaging demonstrates rapid hepatobiliary clearance compared to IgG counterpart

Francis Man, Alexander Koers, Panagiotis Karagiannis_,_ Debra H. Josephs, Heather J. Bax, Amy E. Gilbert, Tihomir S. Dodev, Silvia Mele, Giulia Chiarruttini, Silvia Crescioli, Jitesh Chauhan, Julia E. Blower, Margaret S. Cooper, James Spicer, Sophia N. Karagiannis, Philip J. Blower*

***Corresponding author**: Prof. Philip J. Blower, School of Biomedical Engineering & Imaging Sciences, King’s College London, St Thomas’ Hospital, London, SE1 7EH, UK. Telephone: +44 (0)20 718 89513. E-mail: [philip.blower@kcl.ac.uk](mailto:philip.blower@kcl.ac.uk)

**Supplementary Material**

**Anti-CSPG4 IgE and IgG production**

Anti-CSPG4 IgE and IgG were produced as previously described.^1,2^ Briefly, the coding sequences of the anti-CSPG4 IgE heavy and light chains were cloned into the pVITRO1 vector (pVITRO1-CSPG4-IgE/κ). The anti-CSPG4 IgG vector (pVITRO1-CSPG4-IgG_1_/κ) was generated by linearising pVITRO1-CSPG4-IgE/κ and swapping the constant regions following the Polymerase Incomplete Primer Extension method (Fig. 1). The vectors were used to transfect FreeStyle™ 293-F cells (ThermoFisher). Cells were cultured in hygromycin-containing selection medium and selection-resistant cells, expressing the antibodies, were expanded in serum-free conditions in a 1 L shaker flask (Sigma), 1 L spinner bottle (Sigma) or 5 L WAVE Bioreactor™ system (GE Healthcare). Cell supernatants were harvested after 16 days. The antibodies were purified by affinity chromatography on an ÄKTA Prime system (Amersham) using a pre-packed MEP HyperCel^TM^ column (Pall Corporation) for the anti-CSPG4 IgE and a HiTrap Protein G HP column (GE Healthcare) for the anti-CSPG4 IgG. Purity was assessed by SDS-PAGE using NuPAGE™ 4-12% Bis-Tris gels (Invitrogen).

**Anti-CSPG4 IgE and IgG conjugation to** ***p*-SCN-CHX-A”-DTPA**

Each antibody was first incubated with 50 µM EDTA for 30 min to remove residual metal ions, then washed in 0.1 M HEPES buffer (pH 8.9) and concentrated to 8-10 mg/mL using 30 kDa MWCO Vivaspin® 2 spin filters (Sartorius). A 20-fold excess of [(R)-2-amino-3-(4-isothiocyanatophenyl)propyl]-trans-(S,S)-cyclohexane-1,2-diamine-pentaacetic acid (*p*-SCN-CHX-A”-DTPA; Macrocyclics) at 50 mg/mL in dimethyl sulfoxide (DMSO) was added to the antibody and incubated overnight at 4 °C. The antibody was then extensively washed by spin filtration (as above) in 0.2 M ammonium acetate buffer (pH 6) and concentrated to 2-3 mg/mL.

## ***In vitro* antibody binding**

*In vitro* binding of anti-CSPG4 antibodies to A375 human melanoma cells and U937 human monocytic cells was assessed by flow cytometry and, for A375 cells, immunofluorescence microscopy. To induce expression of FcϵRII (CD23), U937 cells were primed for 24 h with IL-4 (10 ng/mL). Cells were incubated with non-radiolabelled DTPA-analogue conjugated anti-CSPG4 IgE and IgG for 30 min at 4 °C, followed by two washes in PBS + 5 % goat serum. Cells were then incubated with goat anti-human IgE-FITC or IgG-FITC (Jackson ImmunoResearch) (10 µg/mL) for 30 min at 4 °C, washed in PBS + 5 % goat serum and fixed with 1 % paraformaldehyde. Flow cytometry analysis was performed with FACSCalibur™ or FACSMelody™ (BD Biosciences) instruments.

For immunofluorescence microscopy, A375 cells were cultured on 8-well chamber slides (ibidi, #80841) and stained with antibodies as described above. Samples were then coated with ProLong Diamond Antifade mounting media containing DAPI (ThermoFisher) and covered with a glass coverslip. U937 cells were seeded onto poly-L-lysine-coated glass coverslips at 2.5×10^5^ cells per coverslip. After 1 h incubation at 37 °C, the plate was centrifuged, the supernatant was removed and the cells were stained and fixed as described above. After mounting the coverslips onto glass slides with ProLong Diamond Antifade, images were acquired on an Eclipse Ti (Nikon) inverted microscope equipped with a 20× objective and the NIS-Elements control software. Images were processed with ImageJ v1.53c (http://imagej.nih.gov/ij).

**References**:

1. Dodev TS, Karagiannis P, Gilbert AE, Josephs DH, Bowen H, James LK, Bax HJ, Beavil R, Pang MO, Gould HJ, et al. A tool kit for rapid cloning and expression of recombinant antibodies. Sci. Rep. 2014;4(1):5885. doi:10.1038/srep05885

2. Crescioli S, Chiaruttini G, Mele S, Ilieva KM, Pellizzari G, Spencer DIR, Gardner RA, Lacy KE, Spicer JF, Tutt ANJ, et al. Engineering and stable production of recombinant IgE for cancer immunotherapy and AllergoOncology. J. Allergy Clin. Immunol. 2018;141(4):1519-1523.e9. doi:10.1016/j.jaci.2017.12.986

3. Gould HJ, Mackay GA, Karagiannis SN, O’Toole CM, Marsh PJ, Daniel BE, Coney LR, Zurawski VR, Joseph M, Capron M, et al. Comparison of IgE and IgG antibody-dependent cytotoxicity in vitro and in a SCID mouse xenograft model of ovarian carcinoma. Eur. J. Immunol. 1999;29(11):3527–3537. doi:10.1002/(SICI)1521-4141(199911)29:11<3527::AID-IMMU3527>3.0.CO;2-5


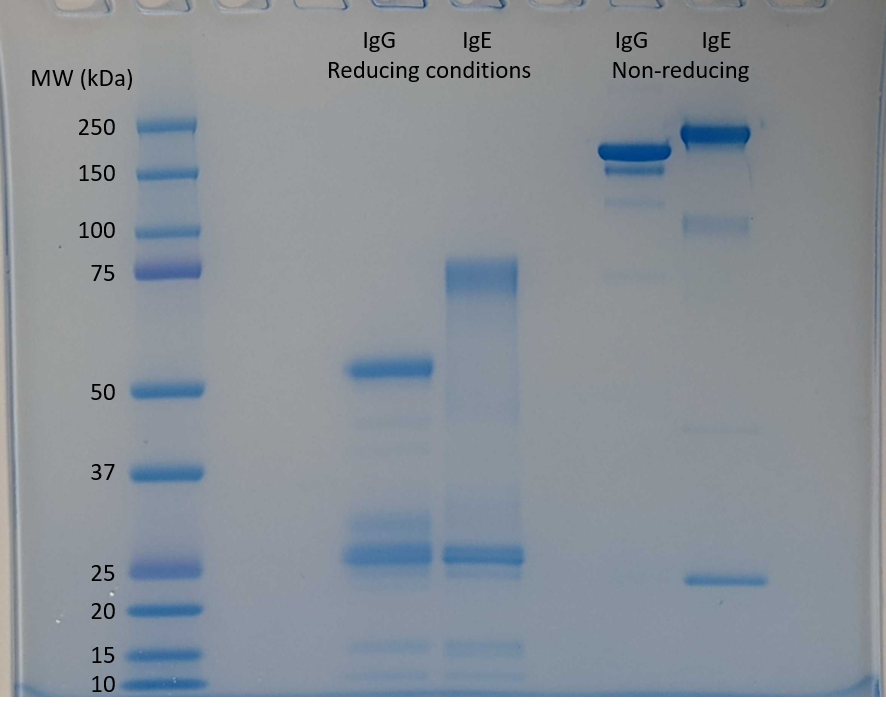


**Figure S1. Characterisation of anti-CSPG4 IgE/G antibody conjugates by electrophoresis.** SDS-PAGE gel (NuPAGE™ Bis-Tris 4-12%, MOPS buffer) of anti-CSPG4 antibodies after conjugation to *p*-SCN-CHX-A”-DTPA. Samples reduced with 50 mM TCEP show bands corresponding to IgG and IgE heavy and light chains, and non-reduced samples show bands corresponding to full-size IgG and IgE.^3^


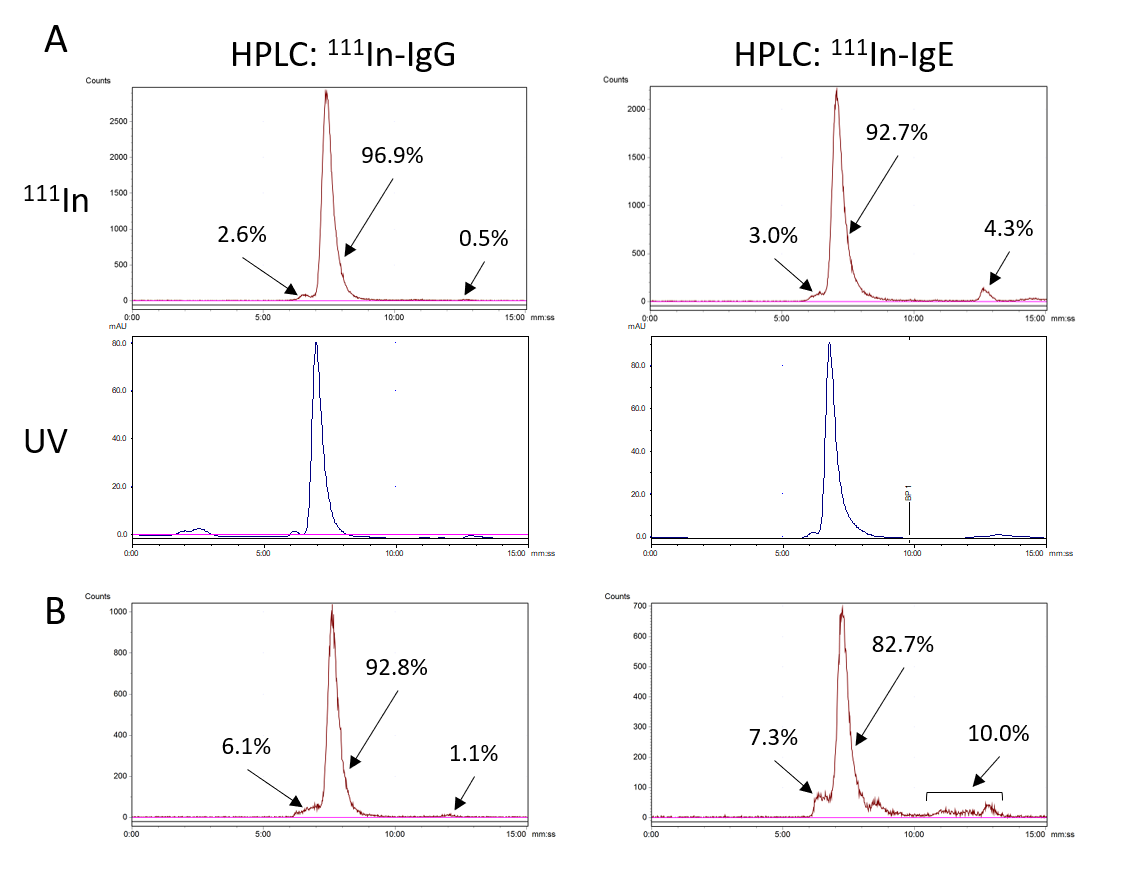


**Figure S2. Antibody radiolabelling: efficiency and stability**. (A) Representative radio-HPLC traces of anti-CSPG4 IgG (left) and IgE (right) radiolabelled with ^111^In (30-31 MBq), showing the γ emissions (top) and UV signal (220 nm, bottom), on a size-exclusion column. (B) Representative radio-HPLC traces of ^111^In-labelled anti-CSPG4 IgG and IgE after 120 h incubation in serum showing the slow appearance of degradation products.


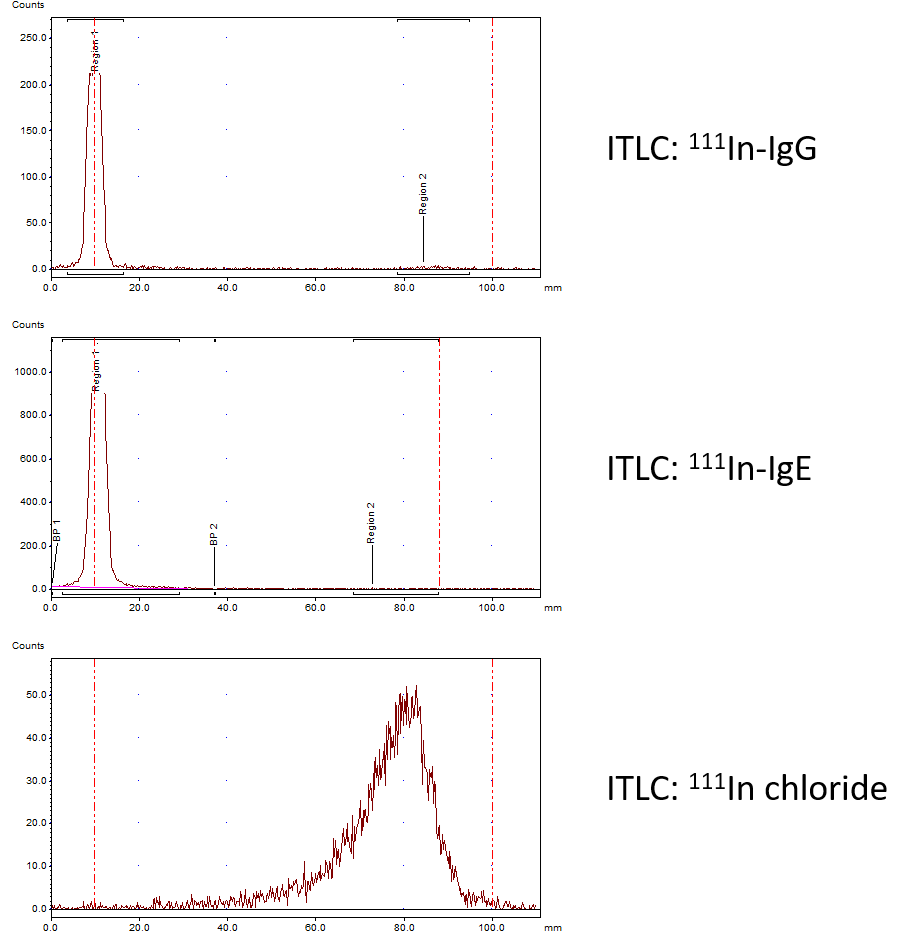


**Figure S3. Antibody radiolabelling efficiency.** Representative radio-TLC traces of anti-CSPG4 IgG (top), anti-CSPG4 IgE (middle) radiolabelled with ^111^In (1-4 MBq), and ^111^In chloride solution (bottom).


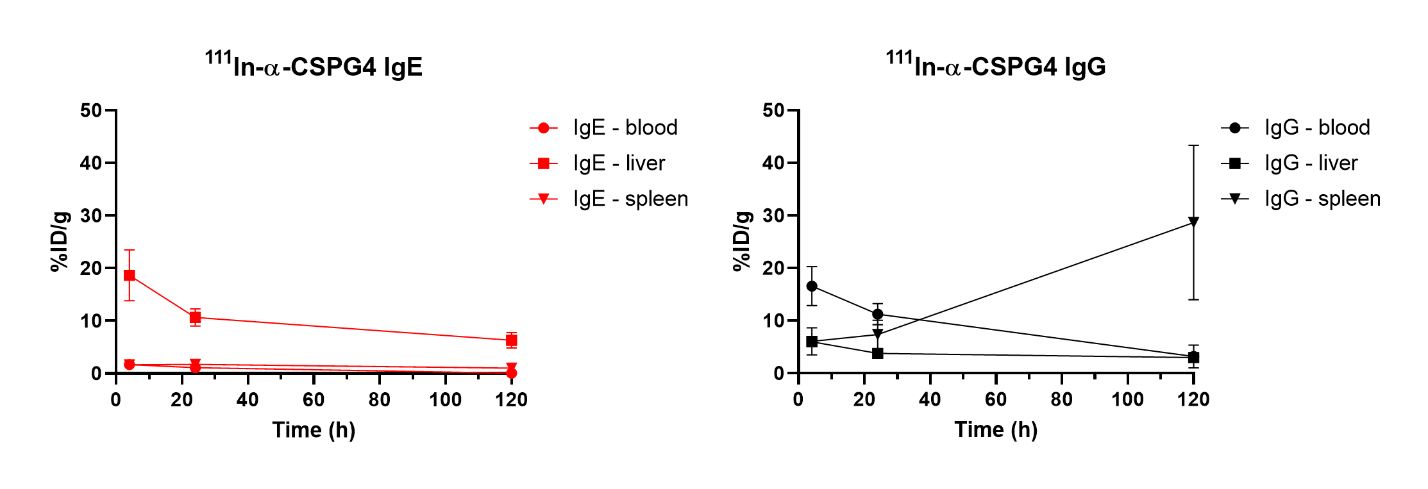


**Figure S4.** Uptake of ^111^In-labelled anti-CSPG4 IgE (left) and IgG (right) in blood, liver and spleen of NSG mice without tumours at t = 4 h, 24 h and 120 h after administration (70 µg antibody, 3-20 MBq ^111^In). Symbols represent the mean±SD of n = 3-4 animals per group.
